# La Grippe

**Published:** 1890-05

**Authors:** E. T. Balch

**Affiliations:** South Bend, Washington


### LA GRIPPE.

E. T. Balch, M. D., South Bend, Washington.

With a zeal and pertinacity of purpose that is so characteristic of Eblis, the wily one, La Grippe came down upon us in full force, attacking right and left, front and rear, sparing neither weak or strong, young or old, rich or poor, saint or sinner—in fact, all grades and conditions of men were alike placed under its malign influence.

But in this highly favored coast country, where zymosis and fevers are almost unknown, where the enigmatical malaria has neither habitation or existence, the scourge was in a great measure robbed of its terrors; so much so, indeed, that very many cases passed safely through the siege, and recovered without any treatment whatever, either lay or professional, and where that damnable drug Quinine, that Moloch of modern scientific stupidity and ignorance, of brutal indifference to human suffering and life, had not been administered, sequelæ and complications have been very rare—mortality almost *nil*—and now La Grippe has left us for pastures new, leaving us the happier for the short acquaintance.

'Twould be tedious and in vain to give more than an outline of the treatment pursued; suffice to say, that every case was carefully individualized, subjective and objective symptoms, mental and physical conditions noted, and especially unusual characteristic symptoms observed; then, and not till then was a prescription made or given according to the law Similia, always in a single dose, and generally in a high potency, and not repeated while improvement continued. Effects were prompt and relief marked in from one to eight hours.

'Tis worthy of notice that blondes suffered at the onset much more severely than the brunettes, although the action of the remedy was quicker, and recoveries more prompt with the former than with the latter.

Then may we not group our cases remedially? as the Acon. case, the Gels. case, and so through all the materia medica. Thus, if an Aconite case is sick from any cause whatever, *Acon.*, and *Acon.* alone is the remedy, the only one that will cure.

With us Acon., Gels., Bry., and Napthalin were most generally indicated. Occasionally an unexpected remedy would come to the front, as Lycopod., and to our astonishment it seemed to cure more rapidly than the generally prescribed drugs.

